# Current-driven fast magnetic octupole domain-wall motion in noncollinear antiferromagnets

**DOI:** 10.1038/s41467-024-48440-9

**Published:** 2024-06-11

**Authors:** Mingxing Wu, Taishi Chen, Takuya Nomoto, Yaroslav Tserkovnyak, Hironari Isshiki, Yoshinobu Nakatani, Tomoya Higo, Takahiro Tomita, Kouta Kondou, Ryotaro Arita, Satoru Nakatsuji, Yoshichika Otani

**Affiliations:** 1 The Institute for Solid State Physics, The University of Tokyo, Kashiwa, Chiba 277-8581 Japan; 2 Center for Emergent Matter Science, RIKEN, 2-1 Hirosawa, Wako, 351-0198 Japan; 3 Key Laboratory of Quantum Materials and Devices of Ministry of Education, School of Physics, Southeast University, Nanjing, 211189 China; 4 Department of Physics, University of Tokyo, Hongo, Bunkyo-ku, Tokyo, 113-0033 Japan; 5 Research Center for Advanced Science and Technology, University of Tokyo, 4-6-1 Meguro-ku, Tokyo, 153-8904 Japan; 6 Department of Physics and Astronomy and Bhaumik Institute for Theoretical Physics, University of California, Los Angeles, Los Angeles, CA 90095 USA; 7 CREST, Japan Science and Technology Agency (JST), 4-1-8 Honcho, Kawaguchi, Saitama 332-0012 Japan; 8 Department of Computer Science, University of Electro-Communications, 1-5-1 Chofugaoka, Chofu-Shi, Tokyo 182-8585 Japan; 9 Trans-Scale Quantum Science Institute, University of Tokyo, Hongo, Bunkyo-ku, Tokyo, 113-0033 Japan

**Keywords:** Spintronics, Electronic and spintronic devices, Information storage

## Abstract

Antiferromagnets (AFMs) have the natural advantages of terahertz spin dynamics and negligible stray fields, thus appealing for use in domain-wall applications. However, their insensitive magneto-electric responses make controlling them in domain-wall devices challenging. Recent research on noncollinear chiral AFMs Mn_3_X (X = Sn, Ge) enabled us to detect and manipulate their magnetic octupole domain states. Here, we demonstrate a current-driven fast magnetic octupole domain-wall (MODW) motion in Mn_3_X. The magneto-optical Kerr observation reveals the Néel-like MODW of Mn_3_Ge can be accelerated up to 750 m s^-1^ with a current density of only 7.56 × 10^10^ A m^-2^ without external magnetic fields. The MODWs show extremely high mobility with a small critical current density. We theoretically extend the spin-torque phenomenology for domain-wall dynamics from collinear to noncollinear magnetic systems. Our study opens a new route for antiferromagnetic domain-wall-based applications.

## Abstract (web summary)

A major advantage of antiferromagnets for applications is the lack of stray fields and insensitivity to magneto-electric perturbations, however, this also makes electric control of AFMs challenging. Here, focusing on a non-collinear AFM, Mn_3_Ge/Sn, Wu et al demonstrate fast domain wall motion, with remarkably low current density, and extend our understanding of spin-transfer torques that drive this to noncollinear antiferromagnetic systems.

## Introduction

Magnetic domain walls, which form between regions of different magnetic domains, are remarkably robust, making them attractive as a potential basis for non-volatile memory devices. The ability to manipulate domain walls electrically is critical to such applications. In ferromagnetic systems, the domain-wall motion can exceed a few hundred meters per second via the intrinsic spin-transfer torque (STT)^1–3^ or the spin–orbit torque (SOT) originating from external spin-current injection^4,5^. However, these high speeds require current densities as high as ~10^12^ A m^−2^, causing inevitable energy loss. Antiferromagnets (AFMs)^6,7^, on the other hand, may provide a new perspective for domain-wall study, where the ultrafast spin-torque driven dynamics potentially speed up domain walls without Walker breakdown and scales down required current densities^8^. In addition, the zero-stray fields of AFMs are favorable for miniaturization and high-density integration. Unfortunately, the zero-stray fields and lack of net magnetization make the efficient manipulation and detection of antiferromagnetic domain walls challenging.

**Fig. 1:**
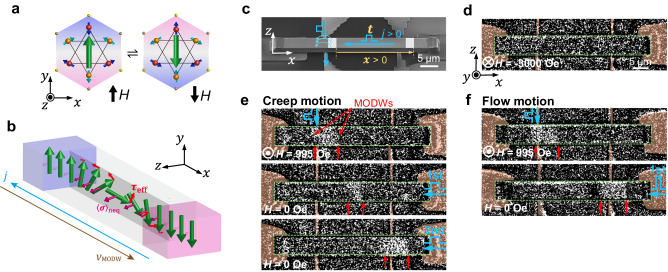
Demonstration of current-driven MODW motion. **a** A pair of oppositely aligned magnetic octupoles reversed by the magnetic field. The green arrows denote magnetic octupole polarizations. **b** The illustration of effective torques (${{{{{{\rm{\tau }}}}}}}_{{{{{{\rm{eff}}}}}}}$) on magnetic octupoles generated by the nonequilibrium spin accumulation (${\left\langle {{{{{\rm{\sigma }}}}}}\right\rangle }_{{{{{{\rm{neq}}}}}}}$) around the domain-wall region. **c** A representative device setup for the current-driven MODW motion. **d** The device MOKE image after the background subtraction. The dashed square depicts the boundary of the sample. **e**, **f** The sequence of the current-driven MODW creep motion under small *j* (**e**) and flow motion under large *j* (**f**). We use the *j* of 9.1 × 10^10^ A m^−2^, 1 ms for the creation. The *j* is set to 1.37 × 10^10^ A m^−2^, 2.5 μs for the creep motion in (**e**), and 6.64 × 10^10^ A m^−2^, 26 ns for the flow motion in (**f**). The red arrows depict the domain boundaries. More representative MOKE images of flow motion can be seen in Supplementary Section 4.

Over the past few years, it has been shown that antiferromagnetic states are electrically manipulable via SOTs in collinear AFMs such as CuMnAs^9^, Mn_2_Au^10^ with inversion symmetry breaking, or noncollinear chiral AFMs Mn_3_X^11,12^ (X = Sn, Ge) with time-reversal symmetry (TRS) breaking. Compared with collinear AFMs, noncollinear Mn_3_X exhibits substantial giant magnetic responses^13–17^ allowing for more efficient detection of the antiferromagnetic state, while retaining the many advantages of antiferromagnetic order. The Mn_3_X hosts a noncollinear antichiral spin structure characterized by cluster magnetic octupole order^18,19^. The magnetic octupole breaks the TRS and is equivalent to the magnetic dipole degree of freedom, reversible under the magnetic field (Fig. 1a).

The current-induced STT given by ${{{{{{\boldsymbol{\tau }}}}}}}_{{{{{{\rm{STT}}}}}}}\propto -\frac{\hslash }{2e}({{{{{{\bf{j}}}}}}}_{{{{{{\rm{s}}}}}}}\cdot {{{{{\boldsymbol{\nabla }}}}}}){{{{{\bf{m}}}}}}$, along with the dissipative counterpart (so called “$\beta$ torque”), is well known for driving domain-wall motion in ferromagnets (FMs) with slowly varying magnetic structure, where ${{{{{\bf{m}}}}}}$ is a unit vector along the localized magnetization direction and ${{{{{{\bf{j}}}}}}}_{{{{{{\rm{s}}}}}}}$ the spin-polarized current density^20^. However, this picture is not directly applicable to the atomistically noncollinear AFMs because of the complex magnetic order. Recent experiments indicate that the electrical current enables switching the magnetic octupole domain^21^ and displacing the magnetic octupole domain wall (MODW)^22^. Nevertheless, how efficiently an electric current can trigger the MODW motion remains a mystery. Moreover, the conventional spin-torque framework calls for a generalization to describe the current-induced spin dynamics with noncollinear texture.

This work addresses the above issues: our magneto-optical Kerr observation demonstrates the current-induced STT can accelerate the Néel-like MODW of Mn_3_Ge up to 750 m s^−1^ with a current density as small as 7.56 × 10^10^ A m^−2^ under no magnetic field (see the Supplementary Video and Supplementary Section 1). The MODW exhibits surprisingly high mobility with a remarkably small critical current density. To shed some light on this, we invoked the *s*–*d* exchange model^23,24^ to investigate microscopically the current-induced STT on MODWs. The nonequilibrium spin density, which is as large as that generated by spin-polarized current in FMs, can induce the effective net torques on magnetic octupoles (Fig. 1b). It motivates us to formulate a general phenomenological framework, which we believe captures the structure of the current-induced coarse-grained dynamics in our (nearly) magnetically compensated noncollinear system. The small net magnetization, which is allowed by crystal symmetries and used to define and probe magnetic octupole domains with conventional means, will be neglected in this phenomenology.

## Results

### Experiments on current-driven MODW motion

To demonstrate current-driven MODW motion, we fabricated micron-sized domain-wall conduits of single crystal Mn_3_X using a focus ion beam (FIB) (Methods). We confirmed the quality of the FIB device by comparing the MOKE hysteresis loops between the bulk and FIB microfabricated samples (Supplementary Section 2). They show the same hysteresis loops, indicating the FIB device keeps the same antiferromagnetic property as the bulk. A representative device structure of Mn_3_Ge is shown in Fig. 1c. The transverse electrode running underneath the conduit without ohmic contact generates a pulsed Oersted field to nucleate a MODW, while the longitudinal terminals are in ohmic contact with the conduit for pulse-current injection. The velocity of MODW is determined by *v*_MODW_ = *x/t*, where *x* and *t* denote the displacement and the pulse duration, respectively. The displacement is captured by a MOKE microscope (Methods). The MODW position and displacement determination are explained in Supplementary Section 3. First, we apply a magnetic field of −3000 Oe to initialize the sample. The background image is subtracted (Fig. 1d) at this saturated state. A pair of MODWs (Fig. 1e, f) are nucleated by combining the transverse pulse-current-induced Oersted field and the perpendicular bias field. After the wall nucleation, a longitudinal pulse is applied to drive the MODW motion without a magnetic field (Fig. 1e, f). The creep motion of MODWs (Fig. 1e) occurs under a small current density *j*, opposite to the electrical current. Meanwhile, the fast flow motion occurs under a large *j* (Fig. 1f) (Supplementary Section 4 for more MOKE images of flow motion). Notably, the domain volume could change after pulse injection (Fig. 1e), possibly due to the extrinsic pinning in the wires (Supplementary Section 5 for more discussion). We estimate the Joule heating due to the pulse-current injection, which increases the temperature by not more than 43 K (Supplementary Section 6 for details). Thus, the sample remains antiferromagnetic during the pulse-current injection. In addition, we examine the magnetization process of the device caused by a static magnetic field for comparison (Supplementary Section 7). The magnetic octupole domain reversal occurs via a reversed domain nucleation at 1000 Oe and the bidirectional propagation of MODWs with increasing the magnetic field until all regions switch at 2100 Oe.

**Fig. 2:**
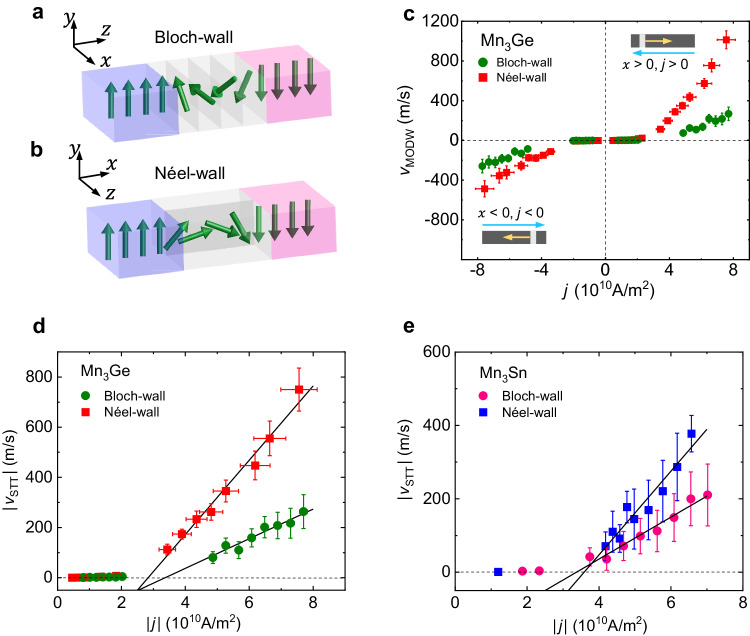
Comparison of Bloch- and Néel-wall velocities in Mn3X. **a**, **b** The illustrations of coexisting Bloch- (**a**) and Néel- (**b**) walls. The dark gray planes represent the kagome planes. **c** The *v*_MODW_ of Bloch- and Néel-walls as a function of the *j* in Mn_3_Ge under both left-to-right and right-to-left displacements. *v*_MODW_ is the average displacement *x* of two MODWs divided by the pulse duration *t* after a single pulse injection. **d**, **e** The |*v*_STT_| of Bloch- and Néel-walls as a function of the |*j*| in Mn_3_Ge (**d**) and Mn_3_Sn (**e**). The error bars (standard deviation) for the velocities are from the multiple measurements of the displacements and the distinguishment of micron-size wall boundaries^41^. Error bars for *j* originate from the nonuniformity of the sample thickness.

For bulk FMs, in general, the magnetostatic energy impedes the Néel walls. Nevertheless, the energetic disparity between the Bloch and Néel walls of AFMs is controlled by crystalline anisotropy due to a significant lack of magnetostatic interaction^25^. Due to the strong in-plane anisotropy, the magnetic octupole moments in Mn_3_X can only rotate in the kagome plane. Thus, the kagome plane orientation distinguishes between the Bloch- and Néel-like MODWs (hereafter referred to as Bloch- and Néel-walls). The directional change in the MODW structure occurs via inter-kagome-plane rotation for the Bloch-wall (Fig. 2a), while intra-kagome-plane rotation for the Néel-wall (Fig. 2b). Figure 2c presents both the *v*_MODW_ of Bloch- and Néel-walls as a function of *j* in Mn_3_Ge for the left-to-right (*x* > 0, *j* > 0) and the right-to-left (*x* < 0, *j* < 0) displacements. We notice an asymmetric variation in the Néel-wall velocity in Fig. 2c. It can be due to the device thickness variation formed during the microfabrication (the left side is about 90 nm thicker than the right in this device, according to the SEM image). Such a wedge shape induces a longitudinal potential gradient, resulting in a unidirectional MODW propagation^22^. Thus, we exclude the nonreciprocal component and extract STT-induced velocity $\left|{v}_{{{{{{\rm{STT}}}}}}}\right|=\,({v}_{+}-{v}_{-})/2$ where ${v}_{+}$ and ${v}_{-}$ denote the left-to-right and right-to-left velocities (Fig. 2d). Similar to FMs, the MODWs exhibit the creep motion in the low current density regime and the flow motion under the high current density regime^26,27^. The critical current density *j*_c_ defined as the horizontal intercept of the linear fitting to the flow-motion regime is 3.37 × 10^10^ A m^−2^ for Bloch-wall and 2.84 × 10^10^ A m^−2^ for Néel-wall. Remarkably, the Néel-wall velocity reaches up to 750 m s^−1^ under a |*j*| of only 7.56 × 10^10^ A m^−2^ under no magnetic field. Similarly, we also measured the |*j*| dependence of both Bloch- and Néel-wall velocities in Mn_3_Sn, as shown in Fig. 2e. The $\left|{v}_{{{{{{\rm{STT}}}}}}}\right|$ of Mn_3_Sn shows the same tendency on |*j*| while having a smaller magnitude than Mn_3_Ge, which can be attributable to the intrinsic difference like band structure^28^ or extrinsic pinning effect. Interestingly, the Néel-wall shows a faster velocity than the Bloch-wall in both Mn_3_Ge and Mn_3_Sn, and we will discuss the possible origin below.

### Theory of current-induced STT in noncollinear AFMs

The current-induced STT can be microscopically pictured through the general *s*–*d* exchange interaction. The nonequilibrium spin density$\,{\left\langle {{{{{\boldsymbol{\sigma }}}}}}\right\rangle }_{{{{{{\rm{neq}}}}}}}$ accumulated near the nonuniform magnetic structure exerts an exchange torque on the localized magnetic moment as^23,24^:

1 $${{{{{{\boldsymbol{\tau }}}}}}}_{{{{{{\rm{ex}}}}}}}=\frac{{J}_{{{{{{\rm{sd}}}}}}}}{{{\hslash }}}\left({{{{{\bf{m}}}}}}\times {\left\langle {{{{{\boldsymbol{\sigma }}}}}}\right\rangle }_{{{{{{\rm{neq}}}}}}}\right)$$

**Fig. 3:**
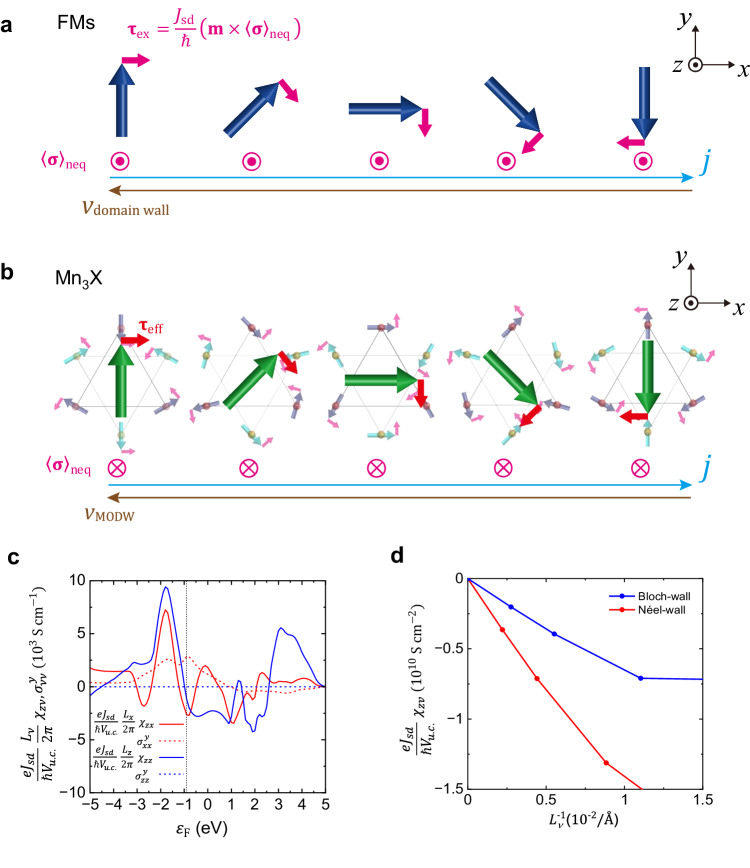
Current-induced STT for MODW motion. **a**, **b** The schematic depictions of current-induced STT for FMs (**a**) and noncollinear AFMs Mn_3_X (**b**). The blue arrows in (**a**) and green arrows in (**b**) denote the localized magnetic moments in FMs and the magnetic octupole moments in Mn_3_X, respectively. The pink arrows describe the electric field-induced ${\left\langle {{{{{\boldsymbol{\sigma }}}}}}\right\rangle }_{{{{{{\rm{neq}}}}}}}$ and the resulting exchange torque ${{{{{{\boldsymbol{\tau }}}}}}}_{{{{{{\rm{ex}}}}}}}$. Note that the effective torque ${{{{{{\boldsymbol{\tau }}}}}}}_{{{{{{\rm{eff}}}}}}}$ (red arrows in (**b**)) on the octupole moment is opposite to the ${{{{{{\boldsymbol{\tau }}}}}}}_{{{{{{\rm{ex}}}}}}}$ on each magnetic moment since the magnetic octupole rotates opposite to each magnetic moment. **c** ${\varepsilon }_{{{{{{\rm{F}}}}}}}$ dependence of ${\chi }_{{zx}}$, ${\sigma }_{{xx}}^{y}$ for the Néel and ${\chi }_{{zz}}$, ${\sigma }_{{zz}}^{y}$ for the Bloch structures in noncollinear AFMs. **d** *L*_*ν*_ dependence of ${\chi }_{z\nu }$ for the Néel-wall (*ν* = *x*) and Bloch-wall (*ν* = *z*) structure. The computational details are given in the “Methods” section.

Here, the ${\left\langle {{{{{\boldsymbol{\sigma }}}}}}\right\rangle }_{{{{{{\rm{neq}}}}}}}=\chi {{{{{\bf{E}}}}}}$ is generated by the electric field **E**, with a response tensor $\chi$. For example, in the conventional FMs depicted in Fig. 3a, the **E** induces ${\left\langle {{{{{\boldsymbol{\sigma }}}}}}\right\rangle }_{{{{{{\rm{neq}}}}}}}$ along the *z*-direction. The torque ${{{{{{\boldsymbol{\tau }}}}}}}_{{{{{{\rm{ex}}}}}}}$ rotates **m** in the clockwise fashion (in the absence of any dissipative effects and anisotropies). Consequently, the domain wall moves opposite to the electrical current. While our treatment will below be generalized to proper dissipative dynamics (where the steady-state motion is established through the balance of the induced dissipative torque and the natural Gilbert damping), these cartoons capture the essential pictures of the domain-wall motion, even in the presence of strong atomistic noncollinearity. For the averaged induced itinerant-electron spin density in Mn_3_X, ${\left\langle {\sigma }_{\mu }\right\rangle }_{{{{{{\rm{neq}}}}}}}={\chi }_{\mu \nu }{E}_{\nu }$, where *μ*, *ν* denote the labels of crystallographic orientations (Methods). Relaxation of the out-of-plane component of this spin density, transferring angular momentum out of the localized orbitals, is microscopically responsible for the dissipative torque, to be discussed below, within the *s*–*d* model. Noticeably, ${\left\langle {\sigma }_{\mu }\right\rangle }_{{{{{{\rm{neq}}}}}}}$ is only finite in the MODW region where there is a gradient of the magnetic structure (Supplementary Section 8). To this end, we apply a single-orbital tight-binding model on the lattice formed by the Mn sites in Mn_3_X (Methods). The Néel-wall (Bloch-wall) is simulated by the antichiral magnetic texture, where each magnetic moment gradually rotates along the *x* (*z*) direction with a period *L*_*x*_ (*L*_*z*_). Figure 3b shows an analogous schematic depiction of the Néel-wall in Mn_3_X case. Similar to FMs, the electric field induces ${\left\langle {{{{{\boldsymbol{\sigma }}}}}}\right\rangle }_{{{{{{\rm{neq}}}}}}}$, which generates torques on each localized Mn magnetic moment. As a result, magnetic octupoles rotate in the clockwise direction. We note that alternative spin-torque mechanisms may arise in a single-layer magnet, which we excluded in our case (see Supplementary Section 9 for details). Figure 3c shows ${\chi }_{z\nu }$ as a function of the Fermi energy ${\varepsilon }_{{{{{{\rm{F}}}}}}}$ for both Bloch- and Néel-wall cases. Here, we only consider ${\chi }_{{zx}}$ for the Néel-wall and ${\chi }_{{zz}}$ for the Bloch-wall case since ${\left\langle {\sigma }_{\mu }\right\rangle }_{{{{{{\rm{neq}}}}}}}$ is confined to the *z*-direction, reflecting the symmetry of the magnetic structures (Methods). Noticeably, both ${\chi }_{{zx}}$ and ${\chi }_{{zz}}$ show complex ${\varepsilon }_{{{{{{\rm{F}}}}}}}$ dependence, which is a clear contrast to the simple sinusoidal curve in the FMs (Supplementary Section 10). However, the magnitude of ${\chi }_{z\nu }$ has the same order as in the FMs, implying that the MODW velocity induced by ${\langle {\sigma }_{\mu }\rangle }_{{{{{{\rm{neq}}}}}}}$ can be as fast as in FMs. Notably, the spin-polarized current has been proposed in the Mn_3_X system, and the spin-polarized current conductivity ${\sigma }_{{jk}}^{i}$ has been defined, where the indices of *i, j, k* denote the directions of spin polarization, spin current, and electrical current^29^. Thus, we also calculate ${\sigma }_{\nu \nu }^{y}$ (*ν* = *x*, *z*) to compare with the ${\chi }_{z\nu }$ both in FMs (Supplementary Section 10) and noncollinear AFMs (Fig. 3c). Our calculation reveals that the ${\varepsilon }_{{{{{{\rm{F}}}}}}}$ dependence of ${\chi }_{z\nu }$ is equivalent to ${\sigma }_{\nu \nu }^{y}$ in FMs but not in noncollinear AFMs anymore. We thus think the spin-polarized current in noncollinear AFMs may not be a good quantity to estimate spin torques for MODW motion. Figure 3d plots a calculated ${\chi }_{z\nu }$ as a function of the period ${L}_{\nu }^{-1}$ (*ν* = *x*, *z*) for Bloch- and Néel-walls (Methods). The ${\chi }_{z\nu }$ is negative in both cases (namely, ${\left\langle {\sigma }_{\mu }\right\rangle }_{{{{{{\rm{neq}}}}}}}$ // −*z* for *j*_*x*_ > 0). Thus, we can expect that the MODWs move opposite to the electrical current in both cases. In addition, $|{\chi }_{{zx}}|$ for the Néel-wall is larger than $|{\chi }_{{zz}}|$ for the Bloch-wall. It may contribute a higher Néel-wall velocity.

The above linear response model is limited in depicting spin torques to the adiabatic process where the dissipative dynamics for domain-wall motion are not covered. To remove this limitation, we have developed a more generic phenomenological framework involving dissipative torques to elucidate MODW dynamics (see “Methods” for details). We parameterize the macroscopic planar octupolar arrangement with angle $\theta \in \left[0,\,2\pi \right)$^19^ and the net nonequilibrium coarse-grained *z*-axis spin density (including both localized and itinerant spins) with $\rho$. The equations of motion deduced from the free energy of the system by recognizing the canonical conjugacy of *θ* and *ρ* are given by:

2 $${\partial }_{t}\theta=\frac{\rho }{{\chi }_{m}}$$

and

3 $${\partial }_{t}\rho=A{\nabla }^{2}\theta -n\lambda \sin \left(n\theta \right)-\alpha s{\partial }_{t}\theta+\tau$$

where *χ*_*m*_ is the (small) spin susceptibility, *A* the (large) exchange stiffness, *λ* the (small) sixfold anisotropy (*n* = 6), *α* the Gilbert damping, *s* the localized saturation magnetization if all spins were hypothetically aligned into a magnetic order, and *τ* the spin torque triggered by the applied current.

The most generic torque associated with planar order-parameter dynamics can be written as:

4 $$\tau=-\frac{{{\hslash }}}{2e}\beta {{{{{\bf{j}}}}}}{{\cdot }}{{{{{\boldsymbol{\nabla }}}}}}\theta$$

where $e$ is the electron charge, ***j*** the applied current density, and $\beta$ a nonuniversal dimensionless coefficient parametrizing a torque that is entirely analogous to the dissipative torques associated with smooth collinear texture dynamics (sometimes referred to as the “nonadiabatic torque” or “$\beta$ torque”). Combining the above torque with Gilbert damping results in the following addition to the bare Hamilton’s equations (i.e., those that follow directly from the free energy; see methods):

5 $${\partial }_{t}\rho \to -\alpha s{D}_{t}\theta$$

where ${D}_{t}\equiv {\partial }_{t}+\eta {{{{{\bf{j}}}}}}\cdot {{{{{\boldsymbol{\nabla }}}}}}$ is a generalized advective derivative parametrized by a material-dependent parameter $\eta \equiv \frac{\hslash }{2{es}}\frac{\beta }{\alpha }$, which has units of the inverse charge density. Note that for $\eta {{{{{\bf{j}}}}}}\sim {{{{{\bf{u}}}}}},$ where ${{{{{\bf{u}}}}}}$ is the drift velocity of electrons (which is generally expected for simple models^30^), $\beta / \alpha \sim s/{s}_{i}$, where ${s}_{i}$ is the (saturated) spin density of the itinerant electrons. The key microscopic modeling challenge is in calculating the value of the parameter *β* (as well as the Gilbert damping *α* in the first place), which is a nontrivial task even in simple ferromagnetic models^31,32^. In particular, while in rudimentary models, $\beta /\alpha \sim s/{s}_{i}$, it is understood to be very sensitive to modeling of disorder and spin relaxation, requiring in practice a systematic diagrammatic treatment^24^. Our work motivates extending this endeavor to noncollinear magnetic systems.

Analyzing the above equations of motion, in the presence of an applied current, we can establish the steady-state velocity ${{{{{\bf{v}}}}}}$ of the MODW by balancing dissipation with the work done by the spin torque. The total power by dissipation and work reads:

6 $$P=- \alpha s {\int {d}^{3}} r \left({{{{{\bf{v}}}}}}{{\cdot }}{{{{{\boldsymbol{\nabla }}}}}}\theta \right)\left[({{{{{\bf{v}}}}}} - \eta {{{{{\bf{j}}}}}}){{\cdot }}{{{{{\boldsymbol{\nabla }}}}}} \theta \left)\right.\right]$$

Setting *P* → 0 gives the steady state:

7 $${{{{{\bf{v}}}}}}=\eta {{{{{\bf{j}}}}}} .$$

We see that the domain wall going faster than this value would lose energy and slow down (*P* < 0), while going slower would gain a net positive work (*P* > 0) into the magnetic texture, thus accelerating the MODW up to the value in Eq. (7). The $\eta$ is understood as the current-driven domain-wall mobility.

**Fig. 4:**
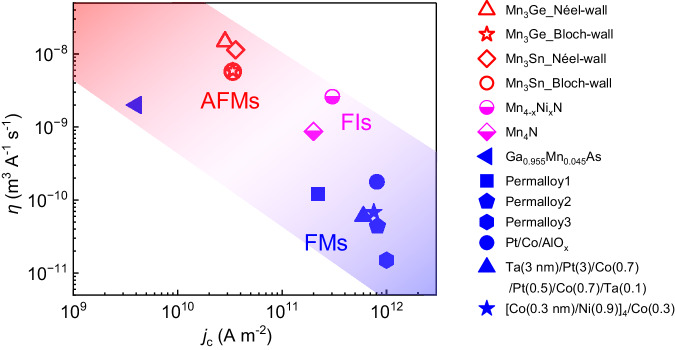
Domain-wall mobilities for various FMs, FIs, and AFMs. The blue, magenta, and red symbols show the FMs (Ga_0.955_Mn_0.045_As^42^, Permalloy1^1^, Permalloy2^43^, Permalloy3^44^, Pt/CoAlO_*x*_^2^, Ta(3 nm)/Pt(3)/Co(0.7)/Pt(0.5)Co(0.7)/Ta(0.1)^45^), [Co(0.3 nm)/Ni(0.9)]_4_/Co(0.3)^46^), the FIs (Mn_4−*x*_Ni_*x*_N^3^, Mn_4_N^47^), and the AFMs (this work), respectively. We note that the *η* and *j*_c_ for permalloy can be different, which is reasonable since the sample condition and composition can differ in various reports.

Lastly, let us discuss more about current-driven domain-wall mobility $\eta$, the magnitude of which depends on materials. Figure 4 plots $\eta$ as a function of *j*_c_ for various FMs, ferrimagnets (FIs), and AFMs obtained from the slopes of *v*–*j* curves. Strikingly, Mn_3_X shows significantly large $\eta$ but rather small *j*_c_. In FMs, $\beta$ torque contributes to both domain-wall depinning and steady motion below Walker breakdown even though it is typically small^23^. On the other hand, the FIs^33^ and collinear AFMs^34,35^ were reported to have much larger *β* torque contributions. Similarly, a significant *β* torque contribution could be accounting for current-driven fast MODW motion.

In conclusion, we have experimentally demonstrated the efficient current-driven MODW motion in noncollinear AFMs, which showcases the significant potential for electrically manipulating the noncollinear magnetic texture. The MODWs show high mobility but small critical current density, even in the absence of a sizeable net spin polarization. For the theoretical analysis, we have extended the conventional STT concepts from collinear to noncollinear magnetic systems. The phenomenology, supported by the microscopic *s*–*d* model, provides insights into understanding the current-induced spin-torque mechanism in noncollinear AFMs.

## Methods

### Single-crystal growth and device microfabrication

Mn_3_X single crystals were synthesized by the Bismuth flux method, the detail of which is shown in our previous work^17^. The Bloch- and Néel-wall devices were prepared using FIB microfabrication (Scios Dual Beam, Thermo Fisher Scientific Ltd.). All the samples in this work have the *y*-direction normal to the surface. The *x*, *y*, and *z* directions correspond to the [$2\bar{1}\bar{1}0$], [$01\bar{1}0$], and [0001], respectively. We first cut out a thin platelet by FIB from the bulk region that shows a clear domain image under 30 kV Ga^+^ acceleration voltage. The sample dimensions are 6 μm in width, 45 μm in length, and ~800 nm in thickness. Before transferring the platelet, we deposited the chromium electrodes onto a Si/SiO_2_ (300 nm) wafer with a thickness of 250 nm using a standard maskless UV lithography and lift-off process. Afterward, the platelet was transferred onto the Si/SiO_2_ wafer on top of the chromium electrodes. We then structured 800 nm tungsten electrodes on both sides of the strip for the longitudinal current injection. After FIB fabrication, the sample was removed and transferred into a milling chamber. We etched the top layer ~40 nm by Argon milling to remove the surface damage, which is well known during FIB fabrication. For example, the damaged layer thickness of the Si surface is about 40 nm for 30 kV acceleration voltage^36,37^. After milling, we immediately transferred the sample to an annealing chamber. We deposited a 3 nm Al_2_O_3_ capping layer to avoid oxidation during the annealing and then performed the annealing to recover the damaged layer on the surface. The annealing temperature was set to 550 °C for Mn_3_Ge and 600 °C for Mn_3_Sn and was kept for 30 min to recrystallize the surface structure. The base pressure of the annealing chamber is ~3 × 10^−7^ Pa and the annealing pressure is about 6 × 10^−7^ Pa.

### MOKE microscope for domain imaging

The domain imaging was carried out by a custom-designed CCD MOKE microscope. A high-resolution (2048 (H) × 2048 (V)) digital CMOS camera (Hamamatsu, C13440-20CU) and an optical objective (×50, Mitutoyo) were equipped for the microscope. We use a mounted LED (Thorlabs, MCWHLP1, wavelength: 400–750 nm) for the light source. The ultimate resolution is 0.13 μm per pixel. Besides, a perpendicular magnet ranging from −7000 Oe to +7000 Oe was set for polar MOKE measurement. The exposure time of the CCD camera was 50 ms. For the current-driven MODW motion, a magnetic field of −3000 Oe was set to saturate the magnetization. After that, a bias field was applied to assist the domain-wall nucleation. The magnetic field was set to zero during the domain-wall propagation. The pulse current is generated by the pulse generator (UTV100, Kentech Instruments Ltd. for nanosecond pulse and WF1948, NF Corporation for microsecond pulse). The measurement was performed in the atmosphere at room temperature.

### Calculation of STT from the *s*–*d* exchange model

To evaluate the electric field-induced torque in noncollinear AFMs, we consider following tight-binding Hamiltonian from the *s*–*d* exchange model on the kagome lattice formed by the Mn sites in Mn_3_X:

1 $$H{{{{{\boldsymbol{=}}}}}}-t\mathop{\sum}_{ < {ia},{jb} > }\mathop{\sum}_{s=\pm }{c}_{{ias}}^{{{\dagger}} }{c}_{{jbs}}+{J}_{{sd}}\mathop{\sum}_{{ia}}\mathop{\sum}_{s,{s}^{{\prime} }=\pm }{{{{{{\bf{m}}}}}}}_{{ia}}\cdot \left({c}_{{ias}}^{{{\dagger}} }{{{{{{{\boldsymbol{\sigma }}}}}}}_{s{s}^{{\prime} }}c}_{{ia}{s}^{{\prime} }}\right),$$

here, *i*, *j* are the labels denoting the lattice vectors, *a, b* ∈ {0,1,2,3,4,5} the sublattice degrees of freedom, $s,s{\prime} \in \left\{+,-\right\}$ the spin degrees of freedom, ${c}_{{ias}}^{{{\dagger}} }$, ${c}_{{ias}}$ the electron creation and annihilation operators, and **σ**_*ss’*_ Pauli’s matrix. The lattice vector **R**_*i*_ is defined by ${{{{{{\bf{R}}}}}}}_{i}={\sum }_{i={{{{\mathrm{1,2,3}}}}}}{n}_{i}{{{{{{\bf{a}}}}}}}_{i}$ with *n*_*i*_ being the integer numbers and **a**_*i*_ the primitive lattice vectors given by ${{{{{{\bf{a}}}}}}}_{1}=\left(A,{{{{\mathrm{0,0}}}}}\right),\,{{{{{{\bf{a}}}}}}}_{2}=\frac{1}{2}\left(-A,\sqrt{3}A,0\right)$, and ${{{{{{\bf{a}}}}}}}_{3}=\left({{{{\mathrm{0,0}}}}},C\right)$. We neglect the breathing anisotropy, i.e., the difference between elementary up- and down-pointing triangles, for simplicity, and the fractional coordinates of the sublattices are set to ${{{{{{\bf{x}}}}}}}_{0}=\left(\frac{5}{6},\frac{4}{6},\,0\right),\,{{{{{{\bf{x}}}}}}}_{1}=\left(\frac{2}{6},\frac{1}{6},\,0\right),\,{{{{{{\bf{x}}}}}}}_{2}=\left(\frac{5}{6},\frac{1}{6},\,0\right),\,{{{{{{\bf{x}}}}}}}_{3}=\left(\frac{1}{6},\frac{2}{6},\,\frac{1}{2}\right),\,{{{{{{\bf{x}}}}}}}_{4}=\left(\frac{4}{6},\frac{5}{6},\,\frac{1}{2}\right)$ and ${{{{{{\bf{x}}}}}}}_{5}=\left(\frac{1}{6},\frac{5}{6},\,\frac{1}{2}\right)$. In Eq. (1), the summation in the first term is restricted to the inter-layer and intra-layer nearest-neighboring sites with the hopping amplitude *t*. The second term *J*_sd_ > 0 describes the exchange coupling between the local and conduction spins. Since **m**_*ia*_ is a unit vector pointing to the direction of the local magnetic moment, *J*_sd_ > 0 means the ferromagnetic coupling of the two spins.

To consider the STT induced by the electric field and the gradient of the magnetic structure, we set ${{{{{{\bf{m}}}}}}}_{{ia}}=({\cos \Phi }_{{ia}},\sin {\Phi }_{{ia}},0)$ with ${\Phi }_{{ia}}={\phi }_{a}^{(0)}+{\phi }_{{ia}}$, where ${\phi }_{a}^{(0)}=\frac{4\pi a}{3}-\frac{\pi }{2}$ describes the inverse chiral magnetic texture and a smooth spatial gradient $\,{\phi }_{{ia}}$. For simplicity, we assume a gradually rotating magnetic structure along the *x* (*z*) direction in simulating the Néel-wall (Bloch-wall) motion. Namely, we set ${\phi }_{{ia}}=2\pi {x}_{{ia}}/{L}_{x}$ ($2\pi {z}_{{ia}}/{L}_{z}$) with ${x}_{{ia}}$ (${z}_{{ia}}$) being the *x* (*z*) axis coordinate of the site specified by $\left(i,a\right),$ and ${L}_{x}$ (${L}_{z}$) denotes the period of the structure. Based on the above, electric field-induced spin density ${\left\langle {{{{{\boldsymbol{\sigma }}}}}}\right\rangle }_{{{{{{\rm{neq}}}}}}}$ is expressed by ${\langle {\sigma }_{\mu }\rangle }_{{neq}}={\chi }_{\mu \nu }{E}_{\nu }$, and the coefficient ${\chi }_{\mu \nu }$ is given at the long-extended lifetime limit $\tau \to \infty$ as follows:

2 $${\chi }_{\mu \nu }{{{{{\boldsymbol{=}}}}}}{{{{{\boldsymbol{-}}}}}}\frac{\pi e}{{N}_{k}} \mathop{\sum }_{k} \mathop{\sum }_{n,m}{{{{\mathrm{Re}}}}}\left[{\sigma }_{k,{nm}}^{\mu }{v}_{k,{mn}}^{\nu }\right]{A}_{{kn}}\left({\varepsilon }_{F}\right){A}_{{km}}\left({\varepsilon }_{F}\right)$$

here, *n*, *m* are the band indices, and *k* is the crystal momentum. The matrix elements ${\sigma }_{k,{nm}}^{a}$ and ${v}_{k,{nm}}^{\nu }$ are given by ${\sigma }_{k,{nm}}^{\mu }=\left\langle {kn}\left|{\sigma }^{\mu }\right|{km}\right\rangle$ and ${v}_{k,{nm}}^{\nu }=\left\langle {kn}\left|{v}^{\nu }\right|{km}\right\rangle$ with the Bloch basis $\left|{kn}\right\rangle$. ${A}_{{kn}}\left(\omega \right)$ is the spectral function for $\left|{kn}\right\rangle$ given by ${A}_{{kn}}\left(\omega \right)=-\frac{1}{\pi }{{{{{\rm{Im}}}}}}[\frac{1}{\omega -{\varepsilon }_{{kn}}+i\eta }]$ where ${\varepsilon }_{{kn}}$ is the dispersion relation and *η* = 1/2*τ* is the smearing width. In the calculation, we set *t* = *J*_sd_ = 1 eV, *A* = 5.66 Å, *C* = 4.53 Å, *ε*_F_ = −0.9 eV, and *η* = 0.1 eV as the model parameters. For comparison, we also calculate ${\chi }_{\mu \nu }$ for the single-orbital square-lattice model with the FM ground state (Supplementary Section 10). In this case, we set *t* = *J*_sd_ = 1 eV, *A* = 1 Å, and *η* = 0.1 eV.

Note that ${\left\langle {{{{{\boldsymbol{\sigma }}}}}}\right\rangle }_{{{{{{\rm{neq}}}}}}}$ should be parallel to the *z*-direction in our setups. In the Néel case, the magnetic structure has the mirror symmetry perpendicular to the *z*-axis, ${\hat{m}}_{z}$, associated with a translation along the *x* axis (Fig. 3b). Thus, due to ${\hat{m}}_{z}{\sigma }_{\mu }=-\!{\sigma }_{\mu }$ ($\mu=x,y$) and ${\hat{m}}_{z}{E}_{x}={E}_{x}$, we find ${\chi }_{{xx}}={\chi }_{{yx}}=0$. In the Bloch case, on the other hand, the structure has the twofold screw symmetry along the *z*-axis, ${\hat{C}}_{2z}$. Since ${\hat{C}}_{2z}{\sigma }_{\mu }=-\!{\sigma }_{\mu }$ ($\mu=x,y$) and ${\hat{C}}_{2z}{E}_{z}={E}_{z}$, we can see ${\chi }_{{xz}}={\chi }_{{yz}}=0$. Thus, in the calculation, we only consider the *z*-component of ${\left\langle {{{{{\boldsymbol{\sigma }}}}}}\right\rangle }_{{{{{{\rm{neq}}}}}}}$ for both cases.

### Theory of current-driven MODW dynamics

We establish the spin-torque framework for MODW dynamics based on the picture from ref. ^19^, by assuming a planar magnetic arrangement, whose local spin order is parametrized by angle *θ* ∈ [0, 2*π*). The dominant exchange interactions result in a noncollinear antiferromagnetic ground state, whose moments fall into an octupolar planar arrangement. The subleading Dzyaloshinski–Moryia interactions define an easy (*xy*) plane for this spin arrangement, reducing symmetry to U(1) spin rotations around *z*. This is further reduced by small ionic anisotropies, resulting in a sixfold anisotropy within the *xy* plane, as well as a small in-plane net canting magnetic moment *m*. *θ* is chosen to be along this moment. The corresponding free energy is:

3 $$F\left[\theta,\rho \right] \, \approx \, \frac{A}{2}{\left({{{{{\boldsymbol{\nabla }}}}}}\theta \right)}^{2}+\frac{{\rho }^{2}}{2{\chi }_{m}}-\lambda \cos \left(n\theta \right)-{{{{{\bf{B}}}}}}\cdot \left(m{{{{{\boldsymbol{\theta }}}}}}+\gamma \rho {{{{{\bf{z}}}}}}\right)$$

where **B** is the applied field, and **θ** = (cos *θ*, sin *θ*, 0). Realistically, a twofold anisotropy is likely induced by growth conditions and/or strain, exceeding *λ*, in which case the above *n* = 6→2. The crystal structure allows for exchange anisotropies and a (very small) *z* magnetization, which we suppress for notational simplicity. In the same spirit, we will focus on the most isotropic and thus generic STT effects, allowing us to restore anisotropies later if needed. According to some arguments and estimations in ref. ^19^, such anisotropies are expected to be relatively small in Mn_3_X.

The equations of motion are obtained from the above free energy in the absence of the applied magnetic field according to the canonical conjugacy of *θ* (a generalized coordinate) and *ρ* (the associated conjugate momentum – being the generator of the *xy* planar spin rotations):

4 $${\partial }_{t}\theta=\frac{\partial F}{\partial \rho }$$

and

5 $${\partial }_{t}\rho=-\frac{\partial F}{\partial \theta }-\frac{\partial R}{\partial \dot{\theta }}+\tau$$

Deriving Eqs. (4) and (5) gives Eqs. (2) and (3) of the Main Text. Here, we have suppressed the magnetic field and added Rayleigh dissipation *R* = *αs*($\dot{\theta }$)^2^*/*2 in the form of the Gilbert damping^38^. Owing to a small spin susceptibility (∝ 1*/A*) suppressing the dynamics of *ρ*, we have only retained the damping of the softest planar variable *θ*. Note that the Gilbert damping results in the relaxation rate *αs/χ*_*m*_ for the out-of-plane spin density. We are not adding current-driven terms to the equation for *∂*_*t*_*θ*, as it is generally expected to result in the subleading effects for low-frequency exchange-dominated dynamics wall study^7^. The above equations are generally nonlinear and should capture both spin-wave properties (with asymptotic sound velocity ${c}=\,\sqrt{A/{\chi }_{m}}$) and domain-wall motion.

The torque *τ* introduced in Eq. (4) of the Main Text matches the “convective” spin transfer discussed in ref. ^19^, corresponding to the overdamped (large *α*) limit of our dynamics, such that ${\partial }_{t}\theta \approx -({\partial }_{\theta }F-\tau )/\alpha s$ (which we will not assume, keeping our discussion more general, although it makes little difference for steady domain-wall motion). Combining our torque with Gilbert damping results in Eq. (5) of the Main Text, parametrized by a material-dependent parameter $\eta$. According to this equation, the current-induced torque amounts in the end to replacing the partial, *∂*_*t*_, with advective, *D*_*t*_, derivatives in the expression for the Gilbert damping. We can understand this heuristically by looking at the *z* polarization of the itinerant electrons (and the associated angular momentum loss) after boosting to the frame of reference co-moving with the drifting electrons. This modifies the spin transfer from the environment into the magnetic system by replacing the bare temporal derivative with the advective one in Gilbert damping. In this picture, we are thinking of Gilbert damping as the main linkage between our magnetic dynamics and the dissipative environment. Since the crystalline environment clearly violates such Galilean invariance, $\eta {{{{{\bf{j}}}}}}\ne {{{{{\bf{u}}}}}}$, in general, while overall phenomenological structure is still maintained (on the basic symmetry grounds). Such arguments have been invoked in the past for current-driven ferromagnetic dynamics^39^, which proved qualitatively insightful in understanding current-driven domain-wall motion^30^.

Analyzing the above equations of motion, in the presence of an applied current, we can establish the steady-state velocity of the domain wall (neglecting any extrinsic pinning effects) by balancing dissipation with the work done by the spin torque:

6 $${\partial }_{t}F={\partial }_{t}\theta {\partial }_{\theta }F+{\partial }_{t}\rho {\partial }_{\rho }F=-\alpha s{\partial }_{t}\theta {D}_{t}\theta$$

Integrating it over space and setting it to zero in the steady state of a domain wall sliding with a fixed profile, $\theta \to \theta ({{{{{\bf{r}}}}}}-{{{{{\bf{v}}}}}}t)$, thus below any kind of Walker breakdown^40^, which we generally do not expect for exchange-coupled antiferromagnetic systems, we obtain Eq. (6) of the Main Text for the total power associated with dissipation and work.

### Supplementary information

Supplementary Information

Description of Additional Supplementary Files

Peer Review File

Supplementary Movie 1

### Source data

Source Data

## Data Availability

The Source Data for the figures of this study are provided with this paper. Source data are provided with this paper.
