# Supplementary material for: Current-driven fast magnetic octupole domain-wall motion in noncollinear antiferromagnets
Source: Nat Commun. 2024 Jun 11;15:4305. doi: 10.1038/s41467-024-48440-9 (PMC11166987; doi:10.1038/s41467-024-48440-9)
Supplement: Supplementary file 1 — Supplementary Information [file 41467_2024_48440_MOESM1_ESM.pdf]

Supplementary information

Current-driven fast magnetic octupole domain-wall motion in noncollinear antiferromagnets

Mingxing Wu, Taishi Chen, Takuya Nomoto, Yaroslav Tserkovnyak, Hironari Isshiki, Yoshinobu Nakatani, Tomoya Higo, Takahiro Tomita, Kouta Kondou, Ryotaro Arita, Satoru Nakatsuji, Yoshichika Otani*

*Corresponding author. E-mail: yotani@issp.u-tokyo.ac.jp

This supplementary information includes:

Supplementary Sections 1 to 10

Supplementary Figs. 1 to 8

Section 1: A representative live imaging of the current-driven MODW motion.

This video records the sequence of the current-driven Néel-wall motion in the microfabricated Mn_3Ge device. The Néel-walls are created by combining the transverse current-induced Oersted field and the applied bias magnetic field. After that, the Néel-walls are shifted by the longitudinal pulse currents step by step. Here, the current density j is set to 9.1×10^{10} A m^{-2}, 1 ms for the creation and 1.37×10^{10} A m^{-2}, 2.5 μs for the creep motion.

Section 2: MOKE hysteresis loops of the bulk and FIB microfabricated samples.

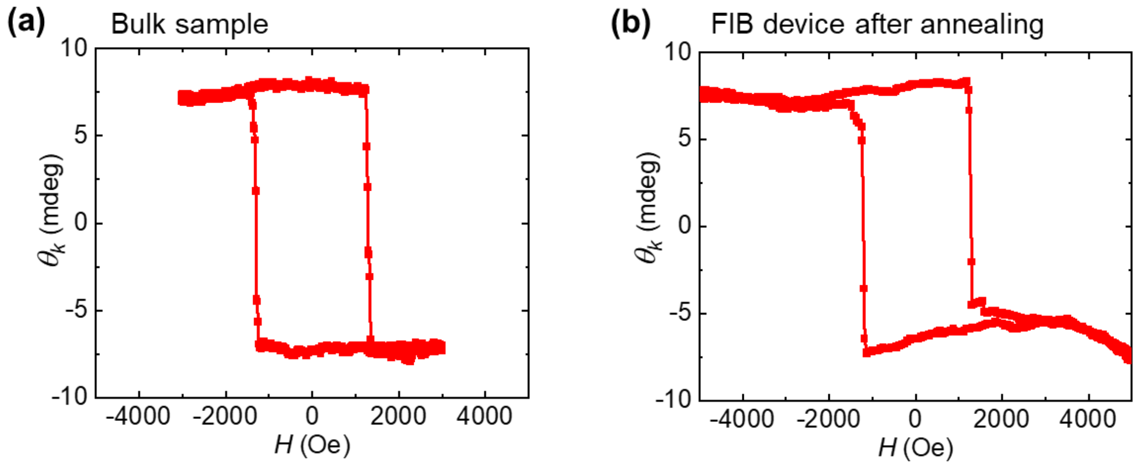

Supplementary Fig. 1 | MOKE hysteresis loops of the bulk and the FIB microfabricated samples. The FIB sample **(b)** shows the same hysteresis as the bulk **(a)**, indicating the FIB sample keeps the antiferromagnetic structure after FIB microfabrication.

Section 3: Determination of MODW position and displacement.

In the experiment, the method of determining the MODW position and displacement is shown in Supplementary Fig. 2. Firstly, we converted the pixel contrast image into the horizontal line profiles and calculated an averaged profile (black line). A smooth profile (red line) was obtained using the adjacent-averaging method. We compared the contrast profile with the raw MOKE image to determine the exact MODW position. The midpoints of the rise and fall lines (The positions of green and blue dashed lines) were defined as the MODW positions. The MODW displacement was calculated by comparing the MODW positions before and after pulse injection. We averaged the displacements of both left and right domain walls for the velocity calculation.

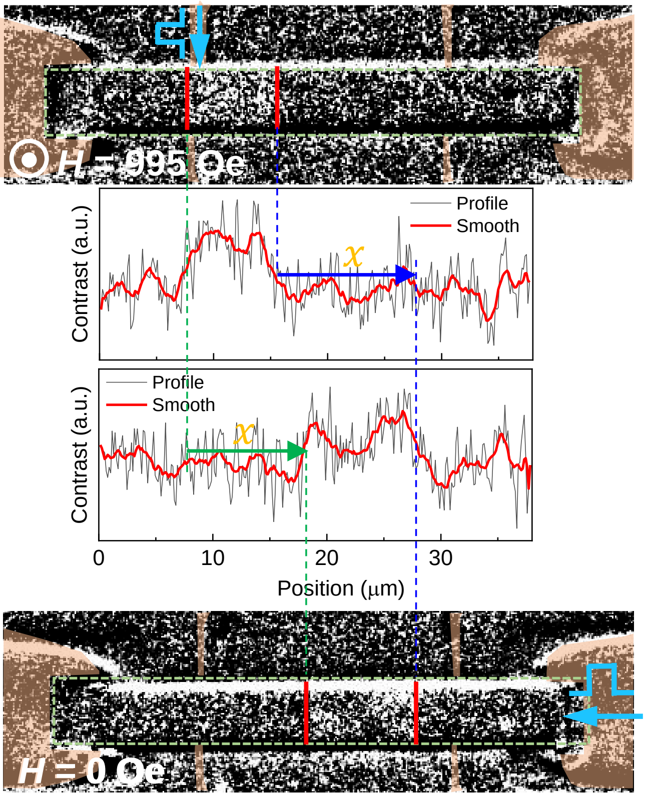

Supplementary Fig. 2 | Determination of MODW position and displacement. The MODW position was extracted from the horizontal contrast profile. The midpoints of the rise and fall lines show the MODW positions. The MODW displacement was determined by two

corresponding MODW positions. The j in this representative MOKW image is set to 3.44×10^{10} A m^{-2}, 100 ns.

Section 4: Representative MOKE images of flow motion.

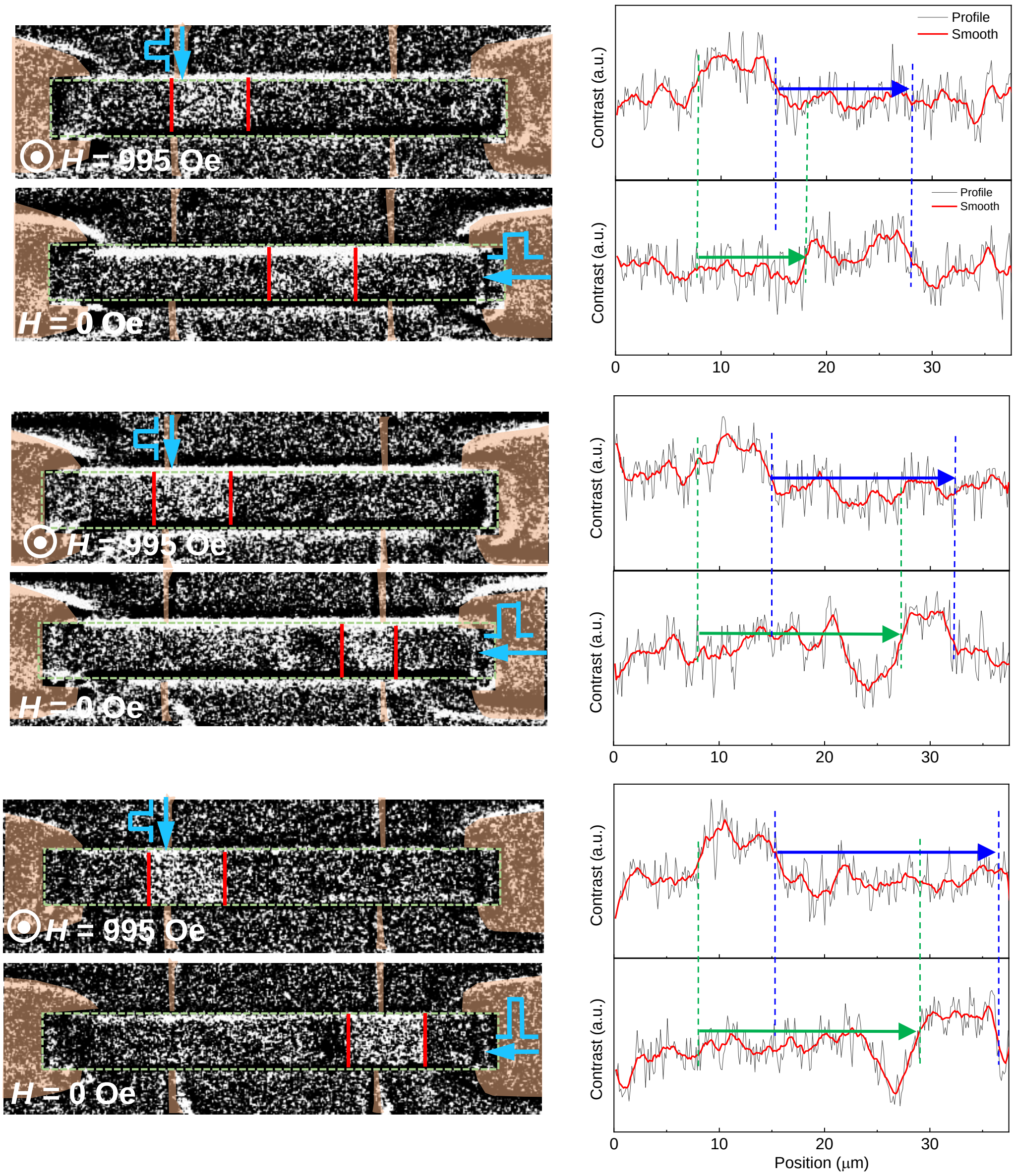

Supplementary Fig. 3 | Representative MOKE images of flow motion. The contrast profiles in the right panel show the MODW positions and displacements. The j is set to 3.44 × 10^{10} A m^{-2}, 100 ns; 4.81 × 10^{10} A m^{-2}, 50 ns; and 6.64 × 10^{10} A m^{-2}, 26 ns, respectively. We checked that the contrast drop around 27 μm remains unchanged under the sweeping magnetic field (Supplementary Fig. 6 in Section 7) and thus should not be the magnetic contrast.

Section 5: Extrinsic pinning effect on MODW motion.

In Supplementary Section 11, we discuss and conclude that the nonadiabatic torque should play a crucial role in MODW motion in noncollinear AFMs. Under this circumstance, the extrinsic pinning effect may affect threshold current density for domain-wall motion and cause an underestimation in velocity[1,2]. We found a threshold pulse duration is required for the MODW motion, similar to the previous report[3]. Therefore, we study the role of Joule heating by comparing the v_{MODW} under different pulse durations in a Mn_3Ge Néel-wall device. The current density is 4.36×10^{10} A m^{-2}. The pulse durations in Supplementary Fig. 4 (b)-(d) are set to 40 ns, 50 ns, and 60 ns, separately. We estimate the v_{MODW} to be 375 ± 16 m/s from (b), 354 ± 58 m/s from (c), and 296 ± 21 m/s from (d). The v_{MODW} is summarized in Fig 4(e). The average velocity from different pulse durations is 342 ± 41 m/s and the error is about 12%. There are two main origins for the velocity variation under different pulses. The domain-wall velocity shows intrinsic stochastic behavior due to the pinning effect. The depinning probability oscillates with the pulse width and depends on the pinning landscape[2]. In addition, the broad domain-wall width of Mn_3X gives rise to another source of velocity uncertainty when determining the domain boundary (e.g., the ~1.4 μm domain-wall width of Mn_3X[4] can cause a velocity difference as large as 56 m/s under a pulse width of 50 ns). Both origins contribute to large error bar in velocities.

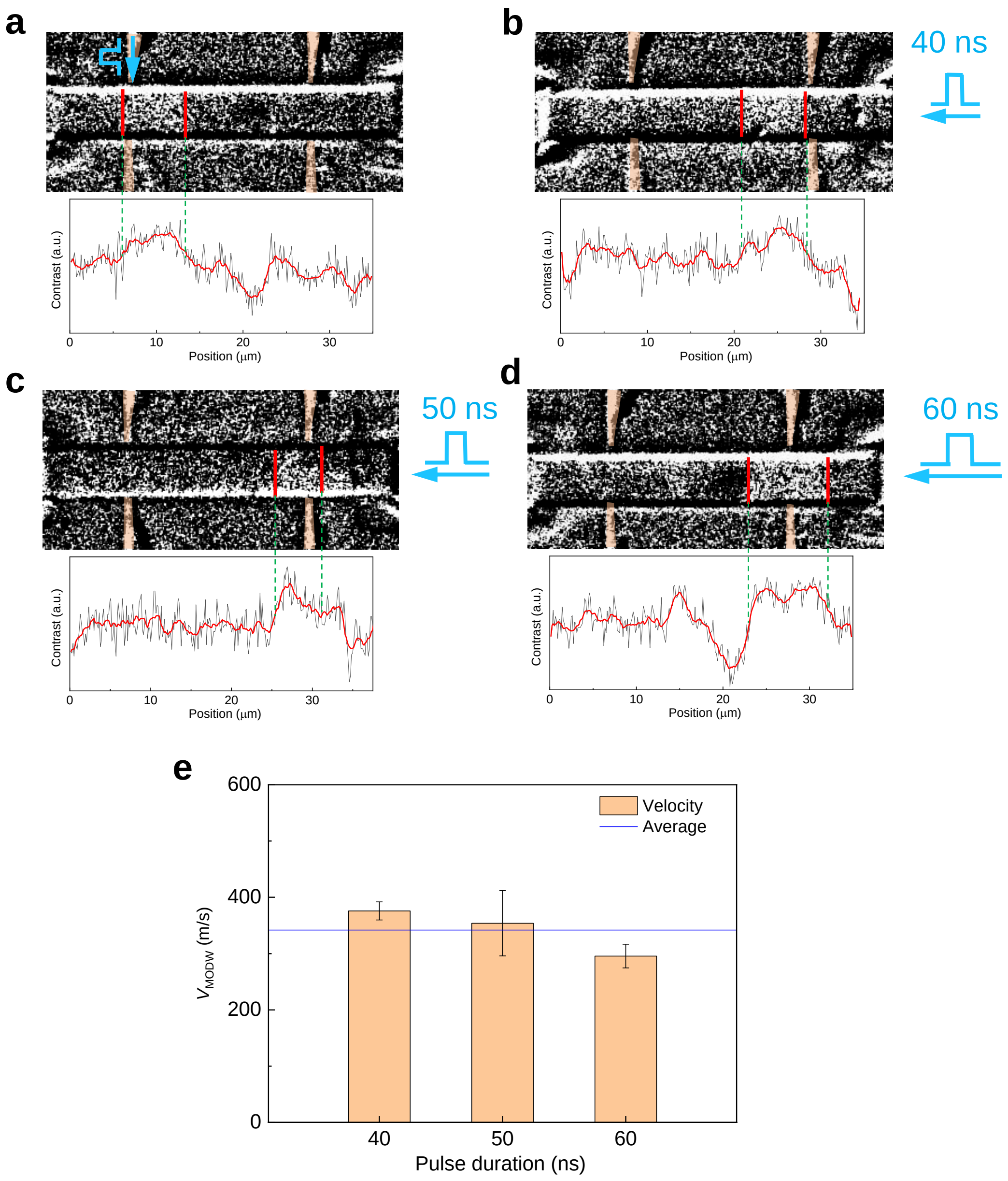

Supplementary Fig. 4 | Estimation of v_{MODW} with different power injection. The pulse durations are 40 ns in (b), 50 ns in (c), and 60 ns in (d). The v_{MODW} is extracted from MODW profiles compared with (a). The right domain wall in (c) tilts after pulse injection. We thus chose the left, middle, and right positions for the velocity estimation, which induces more significant errors in (c). The current density is 4.36×10^{10} A m^{-2}.

Section 6: Estimation of temperature increase due to Joule heating.

Apart from spin torques on the magnetic octupoles, the longitudinal pulse current may also create Joule heating in the sample. We intentionally reduced the pulse duration when increasing the amplitude of current density to diminish the heating effect in the experiment. Joule's Law can estimate the Joule heating generated by the injected pulse current: $Q_g = I^2Rt$, where Q_g, I, R, and t denote the amount of induced heating, applied current, sample resistance, and pulse duration time. On the other hand, the temperature increase can be expressed by the heating accumulation $Q_a = n \cdot C_{v,m} \cdot \Delta T$, where n, $C_{v,m}$, ΔT denote the molar amount, molar heat capacity at constant volume, and temperature increase, respectively. The Q_a is smaller than the Q_g due to the heating dissipation. We can generally neglect the heat dissipation for a nanosecond pulse while not for a microsecond pulse since the temperature gets saturated[5]. Therefore, we ignore the heat dissipation for a nanosecond pulse and have $Q_a \approx Q_g$. Supplementary Figure 5b shows the Q_g obtained from Supplementary Fig. 5a concerning the current density for the nanosecond pulses. Here we use $n = 6.8\times10^{-12}$ mol, $C_{v,m} = 102.2$ J mol^{-1} K^{-1} and $\rho \approx 150\ \mu\Omega \cdot cm$ for the estimation. Ultimately, the evaluated temperature increase is about 43 K, comparable with the previous estimation[6–8]. For microsecond pulses where the heating dissipation cannot be neglected, it reads $Q_a \ll Q_g$. Therefore, we estimated the temperature increase during the pulse injection by monitoring the longitudinal resistance using an oscilloscope. Firstly, we measured the temperature dependence of longitudinal resistance under a small DC current of 50 μA as the reference by heating the device (Supplementary Fig. 5c). When we estimated the temperature increase, the initial resistance $R_0 = 5.51\ \Omega$ before the pulse injection corresponds to the environmental temperature of 275 K in the atmosphere. We note this environmental temperature is anomalously low which may be due to the temperature drift in a cryostat when we measured the *R*-*T* curve. However, this temperature drift does not affect the estimation because it will be cancelled when calculating the temperature change before and after pulse injection. Afterward, we applied a pulse

(9.2×10^9 A m^{-2}, 50 μs), much longer than the experimental value (9.2×10^9 A m^{-2}, 4.5 μs) to get a saturated temperature. We then monitored the resistance change during the pulse injection, as shown in Supplementary Fig. 5d. The spiking peaks originate from the overcompensation of the probe due to the impedance mismatching[9]. The resistance R_t during the pulse injection is not more than 6.14 Ω, corresponding to the temperature of 305 K. The temperature increase due to Joule heating under the microsecond pulse duration is not more than 30 K. The temperature thus remains lower than the Néel temperature (370 K for Mn_3Ge)[10] when the pulse current is applied in our experiment.

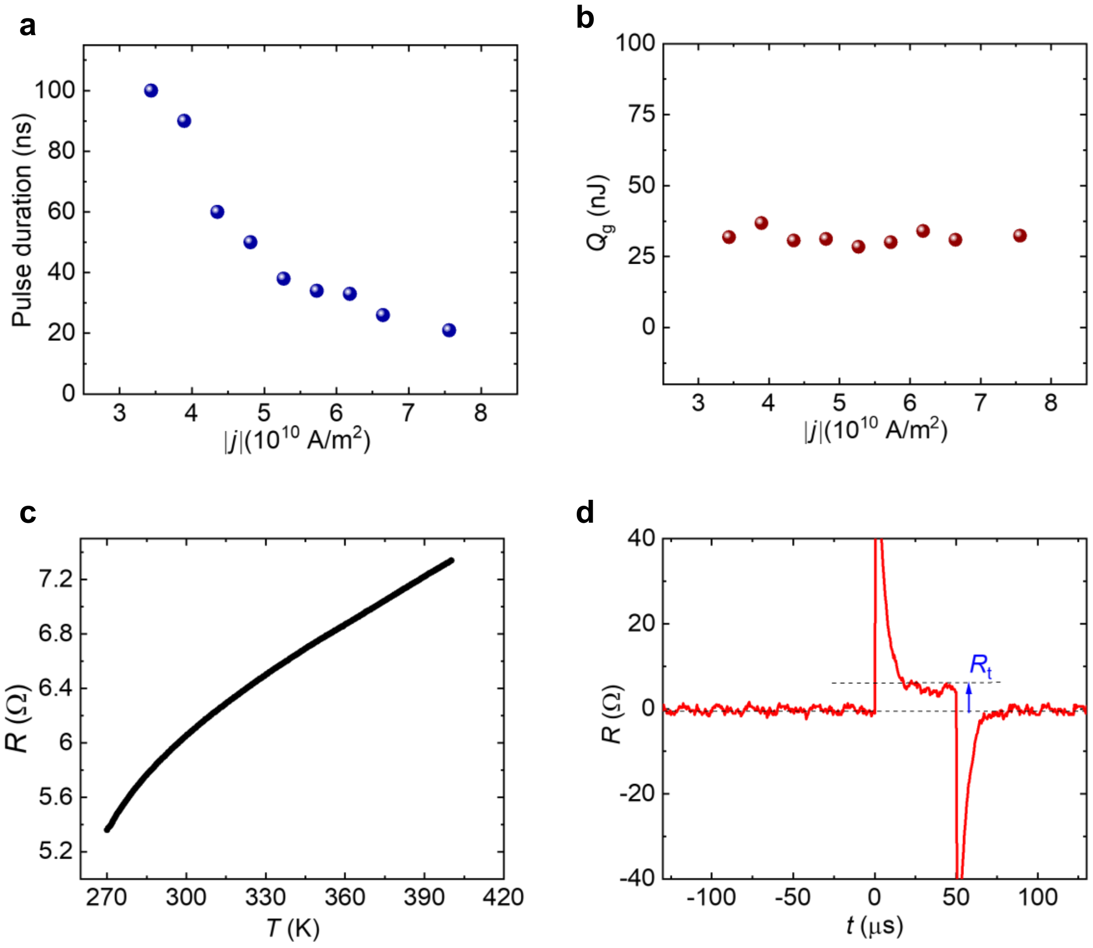

Supplementary Fig. 5 | Estimation of the temperature increase due to Joule heating. a, The pulse duration time for variable current density. **b**, The generated Joule heating Q_g due to the pulse injection. **c**, The temperature dependence of longitudinal resistance under a small

DC current of 50 μA. **d**, The resistance change during the pulse injection monitored by the oscilloscope. The pulse magnitude and duration are set to be 9.2×10^{9} A m^{-2}, 50 μs, respectively.

Section 7: MOKE images of magnetization process under a sweeping magnetic field.

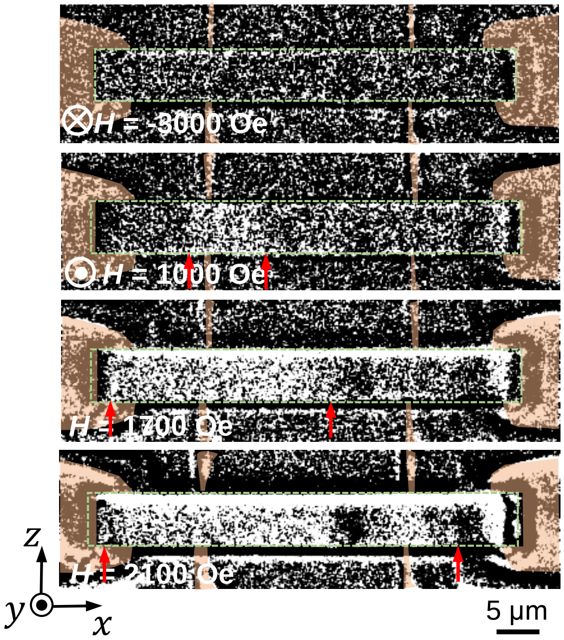

Supplementary Fig. 6 | MOKE images of magnetization process under a sweeping magnetic field. The magnetic octupole domain reversal occurs via domain nucleation at 1000 Oe, followed by MODW propagation with increasing the magnetic field until all the region reverses at 2100 Oe. The MODWs propagate oppositely with increasing the magnetic field. It contrasts the STT-driven MODW motion, where two walls move in the same direction.

Section 8: Sublattice and lattice period L_x dependence of χ_{zx}

Here we demonstrate that both electric field and gradient of the magnetic structure are necessary for finite $\langle\sigma_\mu\rangle_{neq}$. Supplementary Figure 7 shows the sublattice and the lattice period L_x dependence of χ_{zx} for the Néel-wall case. The χ_{zx} for each sublattice remains finite at a large L_x limit ($1/L_x \rightarrow 0$, indicating a uniform magnetic structure). However, the averaged value is zero as expected, which indicates that both finite electric field and gradient of the magnetic structure are required to induce finite $\langle\sigma_\mu\rangle_{neq}$. Thus, the $\langle\sigma_\mu\rangle_{neq}$ is only finite in the domain-wall region where the translational symmetry is broken, and the magnetic gradient is nonzero.

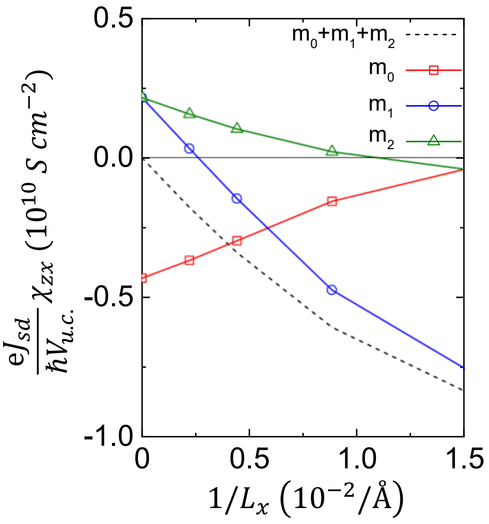

Supplementary Fig. 7 | χ_{zx} as a function of $1/L_x$ and sublattice. The solid lines show the χ_{zx} of each sublattice (m_1, m_2, and m_3). The dashed line shows the χ_{zx} of their summation (m_1+m_2+m_3). We calculated an example of the Néel-wall case at ε_F = -0.9 eV.

Section 9: Discussion of possible spin-torque mechanism in Mn_3X

In addition to the nonequilibrium spin accumulation on the domain wall, there are other current-induced spin-torque mechanisms in a single-layer magnetic device. We thus discuss the possibility in our case. The self-induced SOT at room temperature has been reported in FMs like CoPt[11], FePt[12] and FIs like GdFeCo[13,14], and TbCo[15]. It originates from the concentration gradient-induced inversion asymmetry. Meanwhile, the large spin-orbit coupling of the heavy elements promises a decisive SOT. However, we etched the surface by Ar ion milling up to 40 nm in our FIB microfabricated devices to eliminate the surface damage due to the FIB cut. Thus, the well-ordered hexagonal Mn_3X does not involve the composition gradient. Moreover, the Mn atom has minimal spin-orbit coupling and cannot produce an observable SOT. We exclude the possible self-SOT mechanism in our FIB microfabricated device from these two aspects.

Current-induced nonequilibrium spin accumulation occurs where there is a particular symmetry breaking. Xie *et al.*[16] reported the STT-induced magnetization switching in polycrystalline Mn_3Sn thin film caused by inter-grain spin accumulation where grain boundary breaks the inversion symmetry. Deng *et al.*[17] proposed the interfacial Rashba-induced spin accumulation for the all-electrical switching of polycrystalline Mn_3Sn thin film. However, these two mechanisms do not exist in our bulk single-crystal device. Our experiment used the freestanding single-crystal device, which does not include such inter-grain and Rashba interface-induced spin accumulation. The spin-torque mechanism in this work is entirely different, and we can exclude the above two sources of spin accumulation.

Recent theories revealed that the self-toque occurs in Mn_3X due to the noncollinear magnetic structure[18,19]. However, the difference is that their proposed self-torques correspond to the interface STT while we considered the bulk STT for MODW motion in this work. In Detail, Go *et al.*[18] proposed the sublattice-dependent local spin current in Mn_3X. It can induce the noncollinear torque on the local magnetic moment and switch the magnetic order.

However, the interfacial symmetry breaking is necessary to obtain finite self-torque in their case. Therefore, this type of self-torque is vanishing in our freestanding device. Ghosh *et al*.[19] established a 2D model to analyze the STT in antiferromagnetic tunnel junctions. They found an additional self-generated torque $\mathbf{T}^{\mathrm{SI}}$ besides the anti-damping-like torque $\mathbf{T}^{\mathrm{AD}}$. However, the $\mathbf{T}^{\mathrm{SI}}$ cannot induce deterministic switching because when the magnetic order is rotated, the torque is rotated similarly and can thus never vanish. On the other hand, $\mathbf{T}^{\mathrm{AD}}$ originates from the nonequilibrium spin accumulation, like in our case. However, they expressed $\mathbf{T}^{\mathrm{AD}}$ by the magnetic moments in two magnetic layers separated by a spacer, which differs from our bulk STT. In our case, we considered a uniformly rotating spin texture of cluster octupoles whose gradient is uniform in space. We numerically evaluated a torque acting on each spin based on the microscopic calculation by the Kubo formula. We used an *s*-*d* coupling model without any interface, where we do not have to consider self-induced torques due to the spin current. The calculation shows that the current-induced torques acting on each sublattice in Mn_3X are not fully compensated even though part of them are canceled (Supplementary Section 8). The finite net torques can be a source of the coherent motion of cluster octupole domains. Such calculation of the bulk STT in Mn_3X is essential for understanding the mechanism of STT in a general noncollinear AFM.

Section 10: Calculation of ε_F dependence of χ_{zx} and σ_{xx}^{y} in FMs

We also calculate the ε_F dependence of χ_{zx} and σ_{xx}^{y} in FMs (Supplementary Fig. 8) for comparison to the noncollinear AFMs in Fig. 3c of main text. It is clear to see that χ_{zx} is equivalent to σ_{xx}^{y} and shows a simple sinusoidal function to the ε_F. However, χ_{zx} and σ_{xx}^{y} is more complicated to the ε_F in case of noncollinear AFMs and are not equivalent anymore. It indicates that spin-polarized current is not a good quantity to estimate the spin torques for noncollinear AFMs.

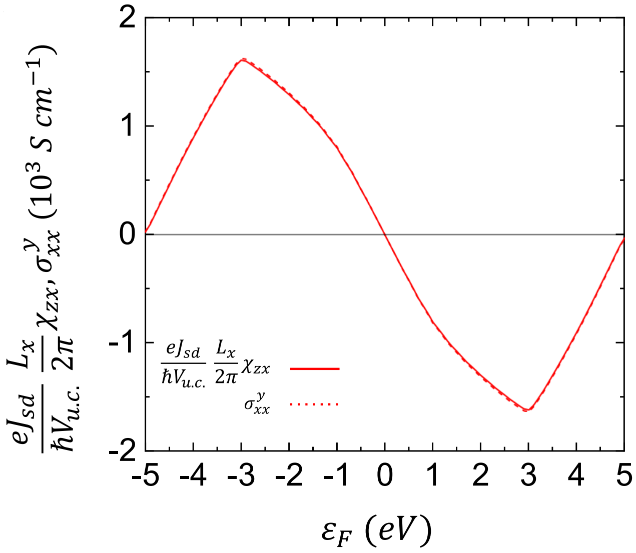

Supplementary Fig. 8 | ε_F dependence of χ_{zx} and σ_{xx}^{y} in FMs. The calculation is based on a single-orbital square-lattice model in ferromagnetic ground state.

Reference

1 Ryu, K. S., Yang, S. H., Thomas, L. & Parkin, S. S. P. Chiral spin torque arising from proximity-induced magnetization. *Nat. Commun.* **5**, 3910 (2014).

2 Miron, I. M. *et al.* Fast current-induced domain-wall motion controlled by the Rashba effect. *Nat. Mater.* **10**, 419–423 (2011).

3 Vélez, S. *et al.* High-speed domain wall racetracks in a magnetic insulator. *Nat. Commun. 2019 101* **10**, 4750 (2019).

4 Wu, M. *et al.* Magnetic octupole domain evolution and domain-wall structure in the noncollinear Weyl antiferromagnet Mn_3Ge. *APL Mater.* **11**, 81115 (2023).

5 You, C. Y. & Ha, S. S. Temperature increment in a current-heated nanowire for current-induced domain wall motion with finite thickness insulator layer. *Appl. Phys. Lett.* **91**, 10–13 (2007).

6 Sugimoto, S. *et al.* Electrical nucleation, displacement, and detection of antiferromagnetic domain walls in the chiral antiferromagnet Mn_3Sn. *Commun. Phys.* **3**, 1–9 (2020).

7 Tsai, H. *et al.* Electrical manipulation of a topological antiferromagnetic state. *Nature* **580**, 608–613 (2020).

8 Takeuchi, Y. *et al.* Chiral-spin rotation of non-collinear antiferromagnet by spin-orbit torque. *Nat. Mater.* **20,** 1364–1370 (2021).

9 Hiscocks, P. D. & Gaston, J. Oscilloscope probes: theory and practice. *Syscomp Electronic Design Limited*. 1–7 (2007).

10 Wu, M. *et al.* Magneto-optical Kerr effect in a non-collinear antiferromagnet Mn_3Ge. *Appl. Phys. Lett.* **116**, 132408 (2020).

11 Liu, L. *et al.* Current-induced self-switching of perpendicular magnetization in CoPt single layer. *Nat. Commun.* **13**, 3539 (2022).

12 Tao, Y. *et al.* Field-free spin-orbit torque switching in L_{10}-FePt single layer with tilted anisotropy. *Appl. Phys. Lett.* **120**, 102405 (2022).

13 Céspedes-Berrocal, D. *et al.* Current-Induced spin torques on single GdFeCo magnetic layers. *Adv. Mater.* **33**, 2007047 (2021).

14 Wang, J. *et al.* Spin-orbit torque in a single ferrimagnetic GdFeCo layer near the compensation temperature. *Appl. Phys. Lett.* **120**, 102402 (2022).

15 Zheng, Z. *et al.* Field-free spin-orbit torque-induced switching of perpendicular magnetization in a ferrimagnetic layer with a vertical composition gradient. *Nat. Commun.* **12**, 4555 (2021).

16 Xie, H. *et al.* Magnetization switching in polycrystalline Mn_3Sn thin film induced by self-generated spin-polarized current. *Nat. Commun.* **13**, 5744 (2022).

17 Deng, Y. *et al.* All-electrical switching of a topological non-collinear antiferromagnet at room temperature. *Natl Sci Rev.* **10**, nwac154 (2023).

18 Go, D. *et al.* Noncollinear spin current for switching of chiral magnetic textures. *Phys. Rev. Lett.* **129**, 1–6 (2022).

19 Ghosh, S., Manchon, A. & Železný, J. Unconventional robust spin-transfer torque in noncollinear antiferromagnetic junctions. *Phys. Rev. Lett.* **128**, 097702 (2022).
