# Supplementary material for: Current-driven fast magnetic octupole domain-wall motion in noncollinear antiferromagnets
Source: Nat Commun. 2024 Jun 11;15:4305. doi: 10.1038/s41467-024-48440-9 (PMC11166987; doi:10.1038/s41467-024-48440-9)
Supplement: Supplementary file 2 — Description of Additional Supplementary Files [file 41467_2024_48440_MOESM2_ESM.pdf]

Title: Supplementary Movie 1
Description: Live imaging of the current-driven MODW motion
